# AN INTERNATIONAL COMPARISON OF INCOME DISPARITIES BY PROBLEM-SOLVING SKILLS, GENDER, AND AGE

**DOI:** 10.1093/geroni/igz038.013

**Published:** 2019-11-08

**Authors:** Nader Mehri, Takashi Yamashita, Roberto Millar, Phyllis Cummins

**Affiliations:** 1 Miami University, Oxford, Ohio, United States; 2 University of Maryland, Baltimore, Maryland, United States

## Abstract

Income disparities by gender have been a persistent problem in economically-developed countries for decades, with income gaps often widening over the adult life course. We use data from the 2012 Program for the International Assessment of Adult Competencies (PIAAC) to examine relationships among problem solving skills in technology-rich environments (PSTRE), income, sex, and age in Australia, Canada, England/Northern Ireland and the United States. Women age 35 to 44 in the middle-to-high (i.e., 50th - 75th percentile) income group had significantly higher PSTRE scores than their male counterparts in Australia and Canada. For the same income group, women ages 55 to 65 had significantly higher. PSTRE scores than men in Canada and England/Northern Ireland. These results suggest that women with similar skills lagged their male counterparts in income in specific sub-populations in specific countries. We provide possible explanations for these differences and conclude with implications for policy and practice.

